# Evaluating the Impact of the Wrapping Bag *TECOtherm Wrabbit* on Temperature Stability During Therapeutic Hypothermia Treatment of Hypoxic Ischemic Encephalopathy

**DOI:** 10.1002/pdi3.70025

**Published:** 2025-09-24

**Authors:** Rashid Katamesh, Katharina Witt, Sebastian Ebert

**Affiliations:** ^1^ Tec Com Medizintechnik GmbH Kabelsketal Germany

**Keywords:** hypothermia, hypoxic ischemic encephalopathy (HIE), neonatology, TECOtherm Wrabbit, therapeutic

## Abstract

During hypoxic ischemic encephalopathy (HIE) therapy for neonates, the patient temperature must be kept at 33.5°C as stable as possible throughout the therapy time. Therefore, hypothermia devices with a fluid‐circulated temperature‐regulating mat have become established. However, therapeutic interventions often require medical personnel to open the mat to reach the patient. This can lead to a loss of thermal regulation due to heat exchange between the patient and environment which can result in a slower or stopped therapy. To mitigate this issue, the wrapping bag *TECOtherm Wrabbit* was proposed to shield the patient from external temperature fluctuations and maintain therapeutic efficacy. We conducted a laboratory experiment using a test model to evaluate the effectiveness of the wrapping bag under two conditions: with and without *TECOtherm Wrabbit*, both under dry and moistened states at room temperatures of 21°C and 27°C. The objective was to assess the retention of patient temperature during possible interventions. Results indicated a significant decrease in temperature when the wrapping bag was not used. Conversely, the use of the wrapping bag demonstrated a slight but controlled temperature change. These findings suggest that the wrapping bag can effectively aid in maintaining patient temperature during necessary clinical interventions. Further clinical research is warranted to validate the application of wrapping bag in maintaining neonatal temperature stability during hypothermic therapy.

## Graphical abstract

Maintaining the patient's temperature at 33.5°C during hypoxic ischemic encephalopathy (HIE) therapy is critical for achieving effective outcomes. Wrapping infants with the *TECOtherm Wrabbit* during therapeutic hypothermia is recommended. This advanced device enhances temperature stability, improves retention during medical procedures, and ensures optimal conditions for successful intervention and improved patient care.

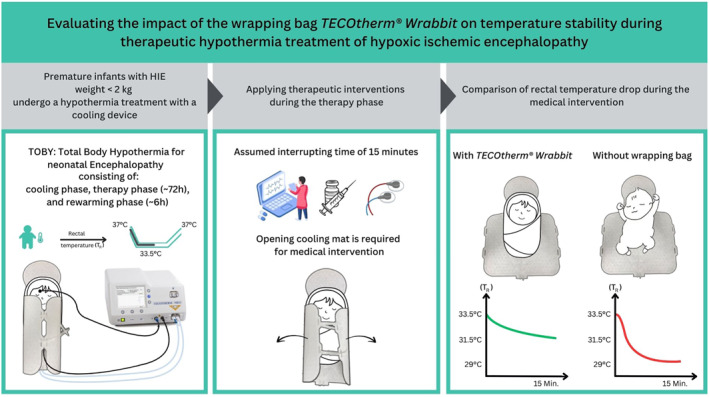

## 1 Introduction

Hypoxic ischemic encephalopathy (HIE) in neonates is caused by oxygen shortage (asphyxia) or blood flow to the brain which leads to brain damage and problems in the neurological system. HIE can lead to detaining neurological development by having a bearing on the central nervous system. One of the available treatments for HIE is the therapeutic hypothermia (TH) [1]. One of the types of TH is surface cooling [2] which passively cools down the core body temperature using a heat exchange mechanism between skin and cooling materials such as a cooling mat. This therapy consists of three phases. The first phase is the beginning phase which cools down the patient to reach body temperature at 33.5°C [1]. The maintenance phase is to keep the body temperature constant at its level for 72 h. The last phase is to progressively rewarm the patient about 0.5°C per hour. This therapy procedure refers to whole body cooling which is called TOBY [3, 4].

TH treatment can be applied to the whole body [5, 6] using a medical device that has a fluid‐circulated mat where the patient is wrapped in. Such a system consists of a central cooling and warming unit that circulates the fluid in the mat through tubes. The device cools or warms the fluid based on the patient's temperature using a rectal temperature sensor which measures the core body temperature. The mat is shaped to cover the whole patient's body and the patient is laid on the mat and its sides are folded around the patient.

Temperature stabilization during the maintenance phase is essential to prevent complications and temperature fluctuation [7, 8]. This is usually achieved by continuous monitoring of vital signs, temperature, diuresis control, and postural adjustment [7]. However, an intervention during the therapy through the nursing or medical team, such as ventilation placing or medicine intravenously delivering requires opening the cooling mat and consequently out‐folding the drape sheet which is according to the instruction of use of the device manufacturer obligatory to use as an insulating underlay between the patient and the mat for protective and safety purposes. Thus, out‐folding the cooling mat and the drape sheet may expose the patient to room temperature which could cause a change in body temperature. The patient’s body temperature may deviate from the set point again as a result of an intervention or loss of device control. Temperature fluctuations can interrupt therapy, whereby an increased temperature can lead to complications or retardation during the treatment. Furthermore, TH using body surface cooling leads to vasoconstriction which lowers temperature control and heat exchange. This can result in ruining the rewarming process [9, 10, 11, 12]. Unregulated rewarming can lead to intracranial pressure rise, electrolyte irregularities, and rebound cerebral edema [2, 4]. Unregulated cooling can result in over‐cooling, which can lead to complications such as cardiac arrhythmia and pulmonary artery pressure [13]. Therefore, it is essential to maintain the patient’s body temperature as steadily as possible during TH therapy.

Different approaches and techniques [14] are discovered to maintain patient temperature in premature infants who are prone to heat loss or hypothermia. One of these techniques is utilizing a hypothermia mat or plastic wraps/bags. Some studies [15, 16] have proved that such techniques can be effective in temperature maintenance or preventing heat loss. Occlusive skin wraps [17] have shown effective thermal protection in infants. Such wraps [18] are utilized to decrease hypothermia in premature infants where they have proven that wrapped infants have improved stability in core body temperature. Other studies [19, 20] have presented that plastic bags for infant bodies except the head can decrease the chance of hypothermia in infants in the delivery room. This indicates that such wraps bags can significantly help by maintaining the core body temperature and reducing heat loss. Abiramalatha et al. [21] have compared many thermal interventions that conserve the temperature for infants in the delivery room such as plastic bags with head covered, thermal mats, plastic bags, or wraps covering the limbs or the torso of the patient. Another study [22] has compared two variants of wrapping plastic bags for conserving heat in neonates.

Throughout the entire cooling therapy, temperature fluctuations during medical interventions must be strictly avoided. This led to the idea of using a wrapping bag in a different form to better preserve body temperature. Instead of using a traditional drape sheet, a specialized version could be designed and shaped similar to a plastic bag that wraps the patient, including the head and limbs, ensuring better insulation and temperature retention.

The objective of this technical study is to experimentally demonstrate that the use of a wrapping bag has a measurable and distinct effect on temperature retention. This will be shown by comparing temperature changes in a test model when using a standard drape sheet that cannot be wrapped around the patient versus a wrapping bag that remains wrapped even when the mat is opened by medical staff.

## 2 Method

Considering the ethical and clinical limitations associated with conducting such a study directly on patients, we initially opted for an experiment with a test model in a controlled laboratory environment where the results can be carefully monitored and analyzed. Using the test model, we can systematically collect data through a series of various test preferences to comprise different cases.

### 2.1 Materials and Equipment

#### 2.1.1 *TECOtherm* NEO [23] and TCmatt [24]

To simulate a real treatment process, a hypothermia/hyperthermia device known as *TECOtherm NEO* was used. *TECOtherm NEO* is designed for hypothermia therapy and features a fluid‐circulated mat known as *TCmatt* that helps regulate the patient's temperature passively. This device can maintain a specific temperature chosen by the user or operate as part of an automated HIE treatment program.

#### 2.1.2 *Raucodrape* and *TECOtherm Wrabbit*

**FIGURE 1:**
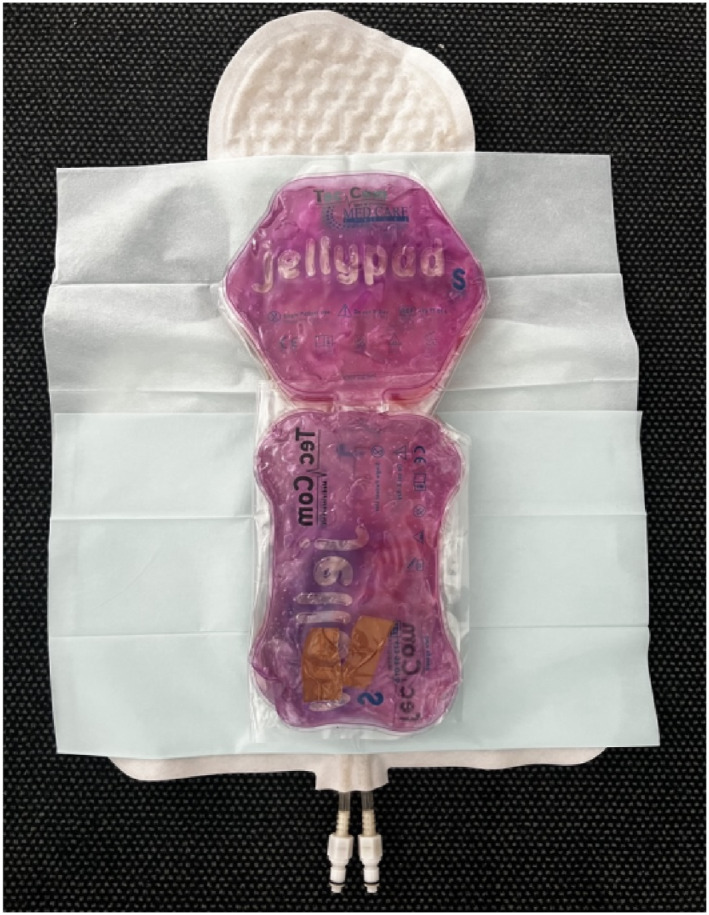
Test model is placed on the hypothermia mat with drape sheet underlay. Drape sheet is considered as safety isolation between patient and mat with no folding mechanism.

*Raucodrape* drape sheet is used as a comparative product as this is currently recommended to be used with the *TECOtherm NEO* as isolating underlay between the patient and the mat (see Figure 1). It has a standard rectangular sheet design and does not incorporate any cap or band mechanisms. Therefore, it does not conform to the patient’s body, and the patient is not directly wrapped in it. *Raucodrape* drape sheet is a product from *Lohmann–Rauscher GmbH & Co. KG* company.

The wrapping bag used in this study is *TECOtherm Wrabbit* [25], where the patient can be completely wrapped in except the face, which remains uncovered. *TECOtherm Wrabbit* is used mainly to protect infants from postnatal heat and moisture loss. By opening one side of the *TECOtherm Wrabbit*, for example, indwelling venous cannula can be applied on the patient without the significant impact of the thermal insulation.

**FIGURE 2:**
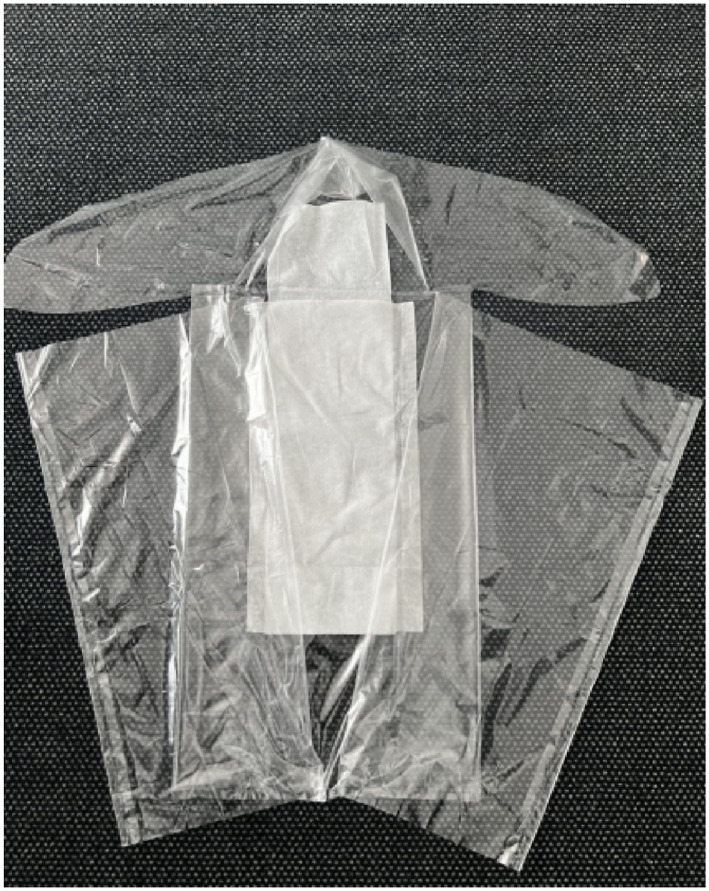
*TECOtherm Wrabbit* wrapping bag.

**FIGURE 3:**
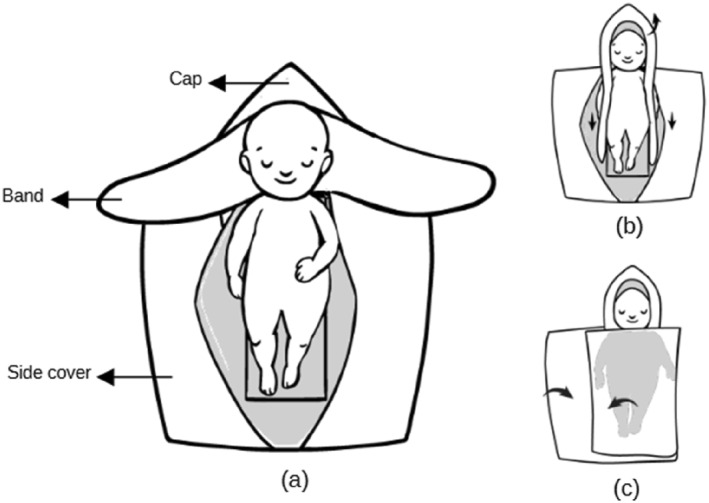
(a) Description of *TECOtherm Wrabbit*, (b) and (c) wrapping the patient.

The used wrapping bag in this study is the *TECOtherm Wrabbit* (see Figure 2). The *TECOtherm Wrabbit* is designed with two side covers, a cap, and side bands (Figure 3a), allowing it to fully envelop the patient (Figure 3c) and covering the head (Figure 3b). It is considered as insulating layer between the patient and the mat. This wrapping bag is produced from polyurethane which is an effective material to help in preserving heat or temperature and hypothermia prevention in neonates [26, 27]. Thus, it is thought to protect the patient against postnatal heat and humidity loss.

*TECOtherm NEO* and *TECOtherm Wrabbit* are products from *Tec Com Medizintechnik GmbH* company, which focuses on producing neonatal medical devices and products.

#### 2.1.3 Test Model

**FIGURE 4:**
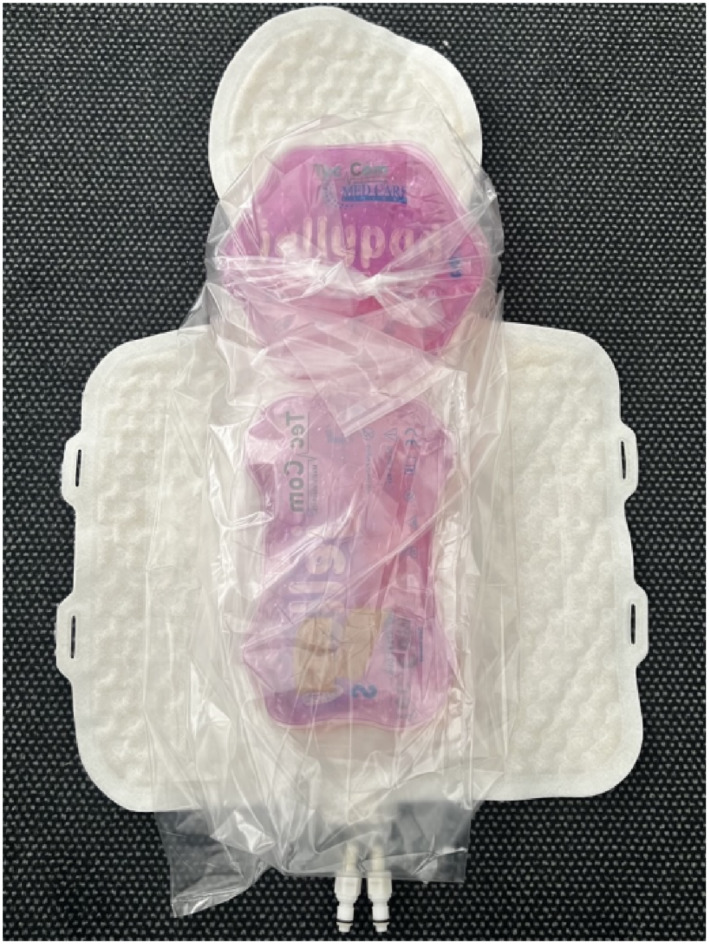
The test model is placed on the hypothermia mat and wrapped in a bag, with only the face partially exposed to room temperature.

As a substitute for a human patient, a test model was developed using hydrogel contained within two plastic bags (see Figures 1 and 4). The primary component of hydrogel is water, which closely mimics the specific heat capacity of the human body (4.18 kJ/kg/K), given that approximately 70% of the human body is composed of water. This means that the amount of heat required to raise the temperature of a given amount of the gel by one degree is comparable to that of human tissue. Hydrogel also contains substances that provide viscosity and thickening.

### 2.2 Test Setup and Environment

For achieving optimal and comparable results, it is obligatory to use the same test conditions and environment. Thus, the experiment is applied in a closed and climatized room with a controlled environment where the room temperature is observed with an external temperature sensor during the whole experiment. The test model is placed on the *TCmatt* either with *TECOtherm Wrabbit* or with *Raucodrape* as insulating underlay.

*TECOtherm NEO* is set up on a patient core temperature of 33.5°C which reflects the optimal temperature in the second phase of HIE treatment. A rectal probe that is connected with the *TECOtherm NEO* is placed between the two hydrogel bags so that *TECOtherm NEO* observes the patient core temperature using the rectal sensor and regulates the fluid temperature based on the targeted patient core temperature of 33.5°C.

An external precision thermometer (manufacturer: *Greisinger*, model: GMH 3710) is positioned near the rectal probe and considered in the experiment to record the patient core temperature (rectal temperature) while it measures more precisely with two decimal digits.

*TECOtherm NEO* is equipped with a skin sensor which is attached to the upper surface of the test model and it is usually used as a reference for the operator to observe the skin temperature of the patient. However, the temperature change of the skin sensor will be also recorded and evaluated when it observes the heat exchange of the surface of the test model with the environment.

The experiment room is temperature‐controlled with air conditioning for 21°C and 27°C, where 21°C is the minimum room temperature of a normal condition and 27°C is the maximum room temperature for operating the *TECOtherm NEO* according to the instruction for use (IFU). In principle, the room temperature was maintained constant throughout the entire experiment, ensuring that the temperature retention observed in the test model during medical interventions can be accurately measured.

Test model condition consists of two states, firstly, the test is run when the model is dry with *TECOtherm Wrabbit* and without *TECOtherm Wrabbit*. The second condition is applied when the test model is moistened with *TECOtherm Wrabbit* and without *TECOtherm Wrabbit*. A water spray is used to moisten the test model. Measured values using the precision sensor and the skin sensor are recorded.

Table 1 summarizes all testing conditions, including the applicability of *TECOtherm Wrabbit*, considered in this experimental study.

**TABLE 1:** Summary of the test cases with all testing conditions.

Test case	Operating room temperature	Test model condition	Wrapping bag
1	27°C	Dry	*TECOtherm Wrabbit*
			None
2	27°C	Moistened	*TECOtherm Wrabbit*
			None
3	21°C	Dry	*TECOtherm Wrabbit*
			None
4	21°C	Moistened	*TECOtherm Wrabbit*
			None

### 2.3 Test Process

After assembling and configuring the test components, the target patient temperature of 33.5°C is set into the device settings of the *TECOtherm NEO* and the therapy will be started. Once the test model reaches the targeted patient temperature and stabilizes at this temperature, the mat is then opened and the temperature reading of the rectal sensor and the skin sensor is recorded during the initial first 15 min of the experiment. The experiment involves only short interventions of maximum 15 min in order to demonstrate the primary effect of using *TECOtherm Wrabbit* on the stability of patient temperature compared to the absence of *TECOtherm Wrabbit*.

## 3 Results

**FIGURE 5:**
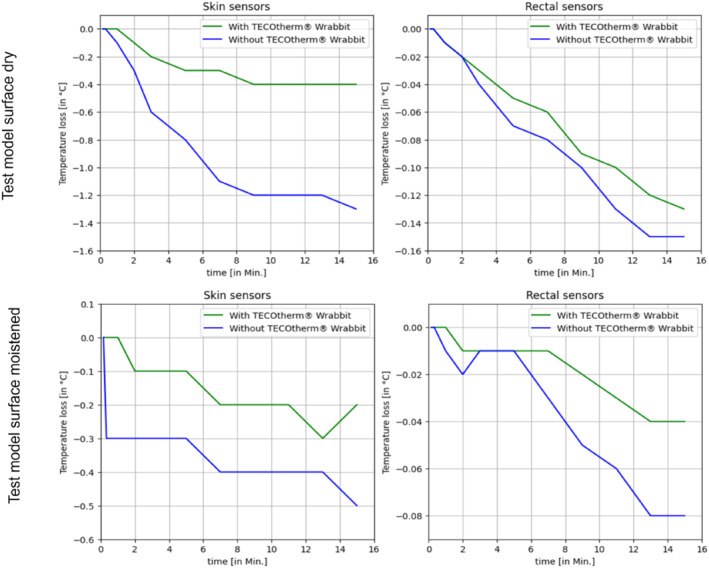
Profile of the temperature loss of the skin temperature probe (left) and the body core temperature probe (right), each with *TECOtherm Wrabbit* applied to the test model (green) and without any hypothermia foil (blue) under dry (top) and moistened (bottom) test model surface at 27°C room temperature.

**FIGURE 6:**
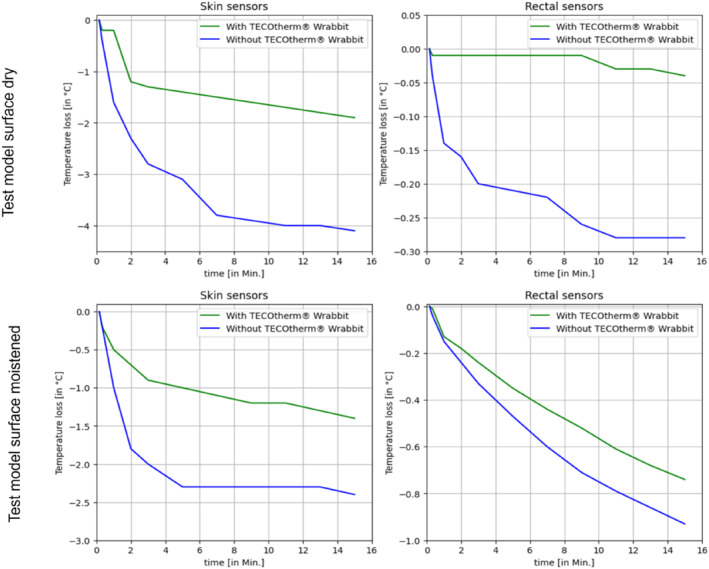
Profile of the temperature loss of the skin temperature probe (left) and the body core temperature probe (right), each with *TECOtherm Wrabbit* applied to the test model (green) and without any hypothermia foil (blue) under dry (top) and moistened (bottom) test model surface at 21°C room temperature.

The objective of this experiment is to test the temperature drop of the test model in a time interval of 15 min after the mat has been opened. The temperature values of the rectal sensor and skin sensor were standardized by calculating the temperature difference which reflects the temperature change. The results of each test case are shown in the figures below which demonstrate the temperature loss of the skin and the rectal sensor when comparing the conditions of applying versus not applying the *TECOtherm Wrabbit* to the test model. The recorded temperature loss, related to the starting temperature, is shown in Figure 5 for 27°C room temperature and in Figure 6 for 21°C room temperature.

In all cases, it is evident that the test model is less affected by a temperature loss with a *TECOtherm Wrabbit* applied to it. This can be seen as well in the rectal sensor and in the skin sensor. The temperature loss of the surface is higher than the core temperature loss and the effect is strongly depending on the room temperature and the moisture the model's surface.

A higher room temperature leads to a less intense temperature loss and a moistened model surface leads to less temperature loss.

Considering the lower room temperature at 21°C, it is remarkable that the greatest rectal temperature loss can be observed when the test model has a moistened surface that is exposed to a low room temperature.

After 15 min, the highest skin temperature loss of −1.9°C with *TECOtherm Wrabbit* attached and −4.1°C without hypothermia mat can be observed at 21°C room temperature with a dry model surface. Under the same conditions with a moistened surface, the surface temperature loss is only −1.4°C with *TECOtherm Wrabbit* attached and −2.4°C without hypothermia mat.

The highest rectal temperature loss after 15 min is −0.74°C with *TECOtherm Wrabbit* attached and −0.93°C without a wrapping bag can be observed at 21°C room temperature with a moistened model surface. Under the same conditions with a dry surface, the rectal temperature loss is only −0.04°C with *TECOtherm Wrabbit* attached and −0.28°C without a wrapping bag.

Table 2 is a summary of the temperature loss of the skin and rectal precision sensor and the temperature difference with and without *TECOtherm Wrabbit* for each test case after 15 min. The device regulates based on the rectal temperature of the test model by using the measured value of the rectal sensor. However, skin sensor indicates the temperature exchange between the environment and the surface of the test model. Therefore, although the rectal temperature is the primary factor influencing the device’s regulation, the skin sensor provides supplementary data regarding the thermal interaction between the environment and the surface of the test model.

**TABLE 2:** Temperature loss (in °C) of skin sensor and rectal sensor complies for different test cases and temperature difference (in °C) with and without *TECOtherm Wrabbit* at 15 min.

Sensor	With *TECOtherm Wrabbit*	Without *TECOtherm Wrabbit*	Temperature difference
Test case 1: 27°C (operating room temperature), dry (test model condition)			
Rectal	−0.13	−0.15	−0.02
Skin	−0.4	**−1.3**	**−0.9**
Test case 2: 27°C (operating room temperature), moistened (test model condition)			
Rectal	−0.04	−0.08	−0.04
Skin	−0.2	−0.5	−0.3
Test case 3: 21°C (operating room temperature), dry (test model condition)			
Rectal	−0.04	−0.28	−0.24
Skin	−1.9	**−4.1**	**−2.2**
Test case 4: 21°C (operating room temperature), moistened (test model condition)			
Rectal	−0.74	−0.93	−0.19
Skin	−1.4	**−2.4**	**−1**

*Note:* Most significant values are highlighted.

The temperature loss of the rectal temperature must be viewed critically as the primary input for the thermal regulation of the *TECOtherm NEO*. Any change in the rectal temperature will lead to an adapted temperature output to counteract the effect. Changes in the surface temperature do not have any input into the thermal regulation. However, skin temperature indicates how heat is exchanged between the patient and the environment. When the patient is exposed to a colder or warmer setting, the skin is impacted first as heat is primarily transferred through the skin’s surface. Ultimately, the use of a wrapping bag on the test model resulted in reduced temperature exchange compared to when the model was unwrapped.

## 4 Discussion

One of the primary treatments of HIE is TH that is applied using a medical device with a cooling mat to lower the core body temperature to 33.5°C for up to 72 h followed by a rewarming phase, during which the device gradually increases the core body temperature back to 37°C. Uncontrolled temperature changes during TH treatment in HIE patients can hinder the benefits of cooling and lead to different health issues and outcome difficulties.

In our experimental study, employing the *TECOtherm Wrabbit,* which can preserve heat or coldness, is investigated to observe and evaluate the temperature change during possible medical interventions. We have examined two situations. Firstly, preserving the core temperature (rectal) at 33.5°C and monitoring the skin temperature while the patient is wrapped in the *TECOtherm Wrabbit* and the second situation is when the patient is not wrapped with *TECOtherm Wrabbit*. The study shows that the *TECOtherm Wrabbit* has played a central role in preserving the skin temperature at an acceptable temperature drop compared to the skin temperature without using a wrapping bag. In general, it could be observed that the skin temperature loss without using a wrapping bag is higher in comparison with the use of a wrapping bag (see Table 2), while the case 2 presents the smallest difference. Test case 2 has the minimum skin temperature difference and this is as a result to the room temperature of 27°C which is closer to 33.5°C compared to 21°C and the test model is moistened which apparently can enhance temperature maintenance and hinder water reduction of the surface of the test model.

Test cases with an operating room temperature of 21°C temperature had a greater temperature drop of the skin and rectal sensors than at the operating room temperature of 27°C. This explains that adjusting the room temperature can affect the test model temperature very strongly. The temperature drop recorded by the rectal sensor shows a stable and gradual decline. Results show that using the *TECOtherm Wrabbit* can slower the core temperature decrease in comparison with no use of a wrapping bag.

There are several restrictions in this experiment. The two selected room temperatures can be marginally different from those found in tertiary or delivery units. Besides, only the first 15 min of the observation are included, during which time actual medical interventions may take longer and the TH device may respond differently. Longer observation time at different room temperatures should be included in future work. The test model used in the experiment was considered to mimic the human body apart from the metabolic heat production or the thermal radiation; however, in order to evaluate the effectiveness of utilizing a wrapping bag during TH therapy, a clinical study involving patients under medical monitoring is essential to validate the potential benefits of using a wrapping bag. To give more precise results of human body interaction with the environment temperature during TH therapy, a bigger patient sample is also needed.

During HIE therapy, any medical or therapeutic intervention that involves in opening the patient’s cooling mat can disrupt the core and skin temperatures. This temperature change can interfere with the therapy as the cooling device will need to readjust the mat temperature to return to the desired level. This process can lead to instability and slow down the effectiveness of the treatment until the temperature is stabilized again. As already mentioned, the uncontrolled rewarming during the TH treatment can lead to complications such as rebound cerebral edema or intracranial pressure rise.

We suggest that, for TH for HIE, the use of the *TECOtherm Wrabbit* which can improve temperature retention during medical interventions that require opening the cooling mat. In comparison to the treatment without the wrapping bag, the results showed a slower and more gradual drop in temperature during the time period of 15 min when the wrapping bag was used. These results demonstrate the potential advantages of utilizing a wrapping bag to avoid issues that could arise during medical interventions, such as losing control of body temperature. To properly evaluate the effect of wrapping bags on temperature regulation during TH in HIE treatment, additional clinical research and observations are needed.

## 5 Conclusion

This technical study provides a laboratory experiment comparing the temperature change in infants during an HIE treatment due to possible medical interventions. It consists of two test conditions: one involving the use of *TECOtherm Wrabbit* and the other without it. The results show retardation of the temperature drop when using the *TECOtherm Wrabbit* compared to a rapid temperature drop when there is no application of *TECOtherm Wrabbit.* These outcomes highlight the importance of using a wrapping bag during HIE therapy and it suggest its potential for temperature retention.

## Author Contributions

Literature search and manuscript preparation: Rashid Katamesh. Study design: Rashid Katamesh, and Katharina Witt. Data interpretation: Sebastian Ebert. Review, supervision, and validation: Sebastian Ebert and Katharina Witt. All authors read and approved the final manuscript.

## Ethics Statement

The authors have nothing to report.

## Conflicts of Interest

The authors declare no conflicts of interest.

## Data Availability

Data sharing is not applicable to this article as no datasets were generated or analyzed during the current study.
